# A scenario of unhealthy life cycle: The role of circadian rhythms in health

**DOI:** 10.1002/agm2.12301

**Published:** 2024-04-09

**Authors:** Manasa Chandramouli, Vrushabendra Basavanna, Srikantamurthy Ningaiah

**Affiliations:** ^1^ Department of Chemistry, Vidyavardhaka College of Engineering Visvesvaraya Technological University Mysore Karnataka India

**Keywords:** aging, carcinogenesis, circadian clock, cryptochromes, unhealthy life‐cycle

## Abstract

Circadian rhythms are oscillations in physiology and behavior caused by the circadian regulator. Cryptochromes, Periods, and Bmal1 are circadian clock genes that have been linked to aging and cancer. Human pathologies alter circadian clock gene expression, and transgenic rats with clock gene defects progress to cancer and age prematurely. In the growth of age‐linked pathologies and carcinogenesis, cell proliferation and genome integrity play critical roles. The relationship concerning the cell cycle regulation and circadian clock is discussed in this article. The circadian clock controls the behavior and countenance of many main cell cycle and cell cycle check‐point proteins, and cell cycle‐associated proteins, in turn, control the activity and expression of circadian clock proteins. The circadian clock can be reset by DNA disruption, providing a molecular mechanism for mutual control amid the cell cycle and the clock. This circadian clock‐dependent regulation of cell proliferation, composed with other circadian clock‐dependent physiological functions including metabolism control, genotoxic and oxidative stress response, and DNA repair, unlocks new avenues for studying the processes of aging and carcinogenesis.

## Graphical abstract

Circadian rhythms have been found to be linked to aging. It was observed that human pathologies alter circadian clock gene expression and causing health issues. This regulates the cell proliferation, metabolism control, genotoxic stress response, and DNA repair and opens new avenues for studying aging and carcinogenesis. The present study, gives a bird's eye view on health effects due to unhealthy life style.

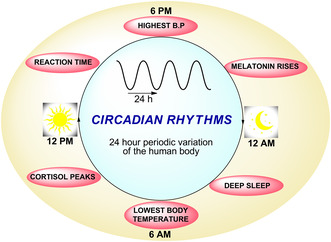

## 1 INTRODUCTION

The earth's rotation about its own axis causes regular environmental deviations that occur in a roughly 24‐hour cycle. Organisms have evolved molecular mechanisms that enable them to quantify time in order to predict regular changes and adapt their physiology accordingly. Circadian clocks are molecular oscillators with intervals that are similar to 24 hours. It is an endogenous mechanism that serves as a timekeeper within the body. Circadian rhythms are 24‐hour oscillations in physiology and action that are caused by the circadian clock.^1^ The ability to adapt to changes in the everyday cycle depends on being able to adjust to the exact environmental 24‐hour rhythm. To reset their period, circadian clocks must be able to integrate external cues.

The circadian clock has many essential functions, like synchronizing multiple biochemical processes throughout an organism and synchronizing an organism to its environment in order to ensure optimum efficiency in various organ systems.^2^ Both prokaryotes and eukaryotes have been shown to have circadian rhythms. The supra chiasmatic nucleus (SCN) is a community of hypothalamic neurons that serves as the command center for circadian rhythms. The SCN synchronizes peripheral oscillator movement, which leads to rhythmic improvements in physiology and behavior.^3^ The circadian production varies by tissue, but it typically involves important functions, including metabolic regulatory pathways, nutrient sensing, mitochondrial activity, intercellular connectivity, cell cycle control, and epigenetic regulation, to mention a few.^4^ The genetic relations among cancer, aging, and the clock may be illuminated by connections among the cell cycle, genotoxic stress response, and the circadian clock.

The circadian clock combines input from the environment's intrinsic state with the organism's intrinsic status. The functional degradation in an organism that raises the risk of disease is referred to as aging. Cancer, metabolic disorders, reduced regenerative ability, and neurodegenerative diseases are all examples. The circadian clock and aging have a bidirectional relationship. This means that a stable aging mechanism necessitates the presence of a functioning timing device. Any aging‐related disorders (such as cancer and metabolic syndrome), have been attributed to a higher risk of cancer. In the human body, aging is associated with a reduction in body fat and a rise in physical activity.^5^ The latest state of understanding regarding the mammalian circadian clock and aging is discussed. We also look at how reviewing circadian disturbance can be used as a model for studying aging processes.

## 2 THE MOLECULAR CLOCKWORK IN MAMMALIAN ORGANISMS

**FIGURE 1:**
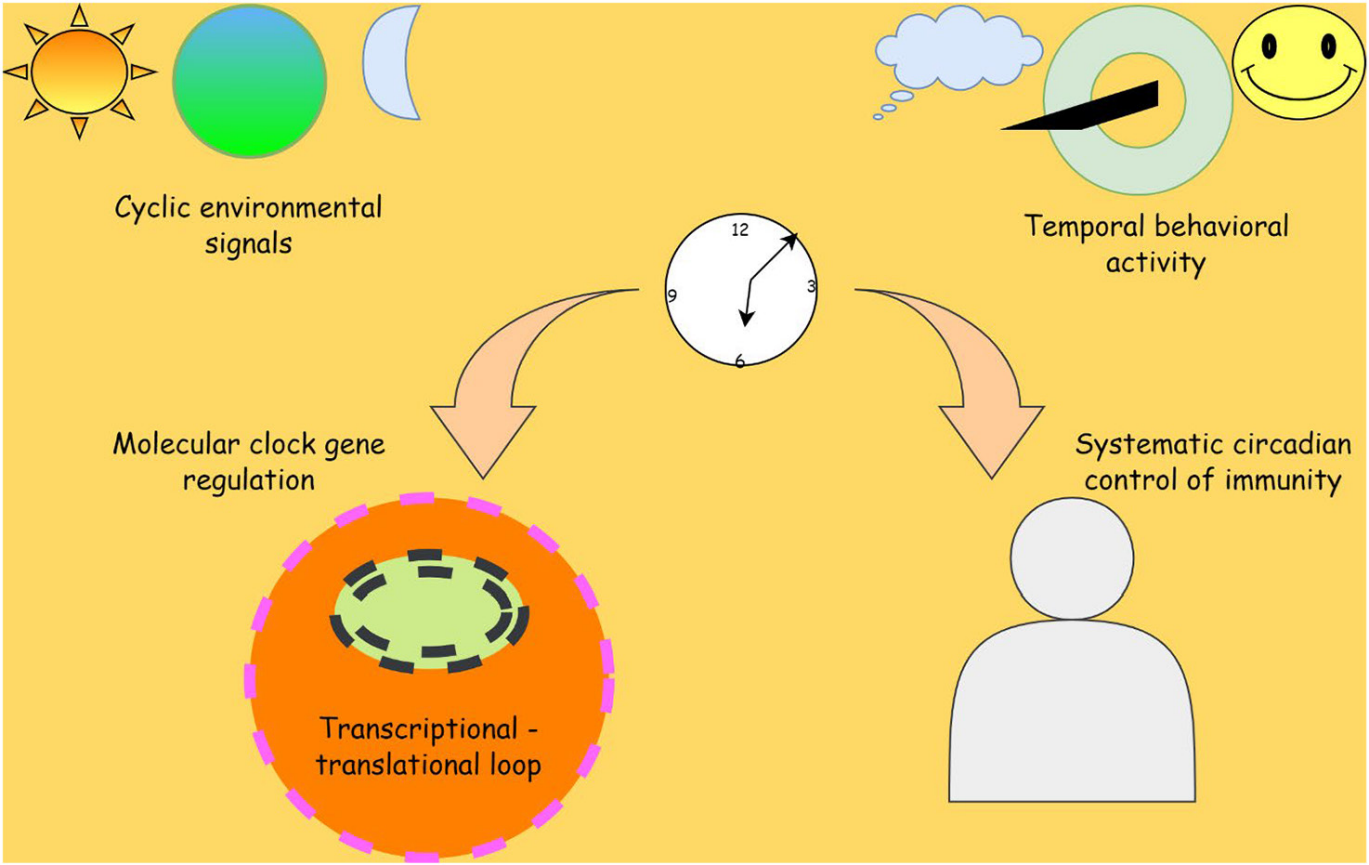
Implication of the circadian clock in cell cycle regulation‐operation of the central circadian clock.

The transcriptional/translational feedback loops that result in the operation of the central circadian clock make up the mammalian clock, as shown in Figure 1. Period (Per) and cryptochrome (Cry) genes, which are transcriptional targets of PER and CRY genes, are among the clock's targets.

The transcriptional loop of the clock is formed by the transcriptional receptors Nr1d1d and Nr2, as well as RAR elements and Bmal1 expression (RORs). The time and Cry activate the clock, inhibiting BMAL1/CLOCK transcriptional function and resulting in decreased transcription.^6^ BMAL1/CLOCK controls the oscillatory transcription of several clock‐controlled genes that determine clock production, in addition to regulating the expression of genes in the core loop and stabilizing loops. The high tissue specificity of a clock's operation, on the other hand, is dependent on interactions among the core clock and tissue‐specific transcription factors and epigenetic regulation.^7^ The Center for Cancer Genomics have a complex regulatory system that allows for transcriptional oscillations at different times of the clock.^8^ Rhythmic transcriptional oscillations of particular target genes are produced by the core transcriptional clock machinery. RNA degradation regulates circadian oscillations at the RNA level as well. Other non‐clock‐dependent pathways also play a role in tissue physiology's temporal organization. To comprehend regulation, we must first comprehend the entire scope of cellular and tissue regulation to have a better understanding of cellular, tissue, and organ regulation.

## 3 THE CIRCADIAN CLOCK NETWORK'S SYSTEMIC CELL CYCLE ORGANIZATION

The inward photic information is converted to chemical information in the SCN, which “sets” the process of gene expression in individual SCN neurons. SCN oscillations are tightly coordinated, thanks to strong neurotransmitter‐mediated coupling among neurons. The SCN is required for the circadian rhythmicity of loco motor development, endocrine activity, endocrine drinking and eating, and a variety of other physiological processes. For mice kept in full darkness, some SCN‐lesioned mice have confirmed a lack of circadian rhythms in peripheral tissues.^9^

**FIGURE 2:**
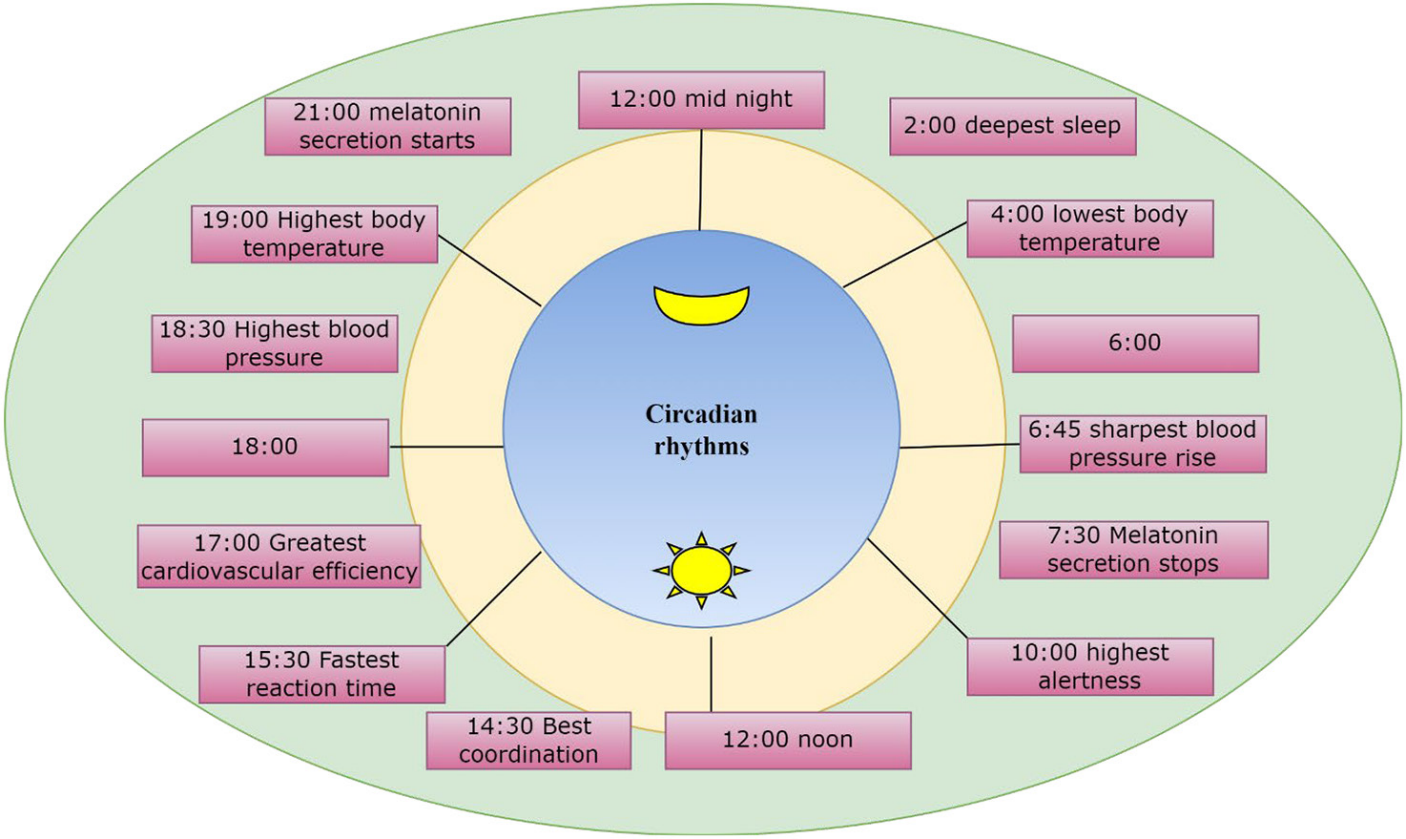
Regular circadian rhythms’ performance of the circadian clock.

Several studies have identified inconsistencies on the issue of whether the SCN is relevant for determining whether the mouse is eating or drinking. With the exclusion of the testis and thymus, almost every cell shows the ability to express the central circadian circuitry, and virtually all peripheral tissues exhibit circadian rhythms.^10^ In either case, the SCN has the ability to mediate synchronization of peripheral tissue clocks through mechanisms that are likely to work in tandem. Via processes, such as temperature cycles and feeding patterns with their cycles, the SCN will indirectly transfer time of day information to peripheral tissues.^11^ Several aging‐related signaling pathways are intertwined with this central clockwork. The relationship of the clockwork with these factors will change as people age, influencing the performance of the circadian clock^12^ (Figure 2). The circadian transcriptome reprograming of the transcriptome, which controls several clock‐controlled genes in a tissue‐specific manner, helps to slow down the aging process.^13^ Dietary restriction, which inhibits both of these nutrient identifying conduits as well as circadian clock activity, can also affect the circadian clockwork.^14^

The cell cycle's regulation emerged as a defense mechanism in contradiction of DNA‐damaging UV radiation. There are cells in both the quiescent and proliferative stages in almost every tissue.^15^ The circadian clock and the cell cycle, we suggest, are two periodic cycles that are difficult to distinguish in space. It is a focus that should be encouraged on recent research on the role of the circadian transcription factors BMAL1 and CLOCKOULDER in cell cycle regulation.

## 4 THE SYSTEMIC CIRCADIAN CLOCK AND AGING: MOLECULAR CONNECTIONS

It is likely that peripheral tissues have an additional layer of synchronizing connectivity. Peripheral tissues and the clock are synchronized by the SCN and/or the clock. Aging‐related improvements to the input pathways, the SCN, and the circadian clock at the periphery may thus affect the circadian clock's robustness. The circadian clock network is affected by aging on many levels. As a consequence, the SCN's capacity to react to light is reduced due to decreased sensitivity to photic feedback, intercellular binding, and signal transmission.^16^ The loss of amplitude of neurons and a reduction in the number of neurons in the aged SCN affect the activity of the aging‐related clock genes. The absence of neurons has a significant effect on both the systemic and human SCN levels. To maximize the longevity of hamsters, it is most likely based on SIRT1 and SIRT2, as well as a young donor into an elderly recipient.^17^

The aging of an SCN is similar to the aging of the human brain in that it is caused by a sequence of events that result in a decrease in the ability of neurons to respond to light properly and a loss of the ability to send signals to the brain. The lack of light sensitivity, decreased expression levels, and a reduction in the number of neurons are all symptoms of this.

Behavioral cycles, such as the sleep–wake cycle and physical exercise, deteriorate as people become older. Exercise, for example, improves sleep patterns, has an effect on sleep cycles, and aids in the entrainment of the circadian clock. With a transition in circadian activity, the amplitude of the neural SCN output, as well as the humoral output of the SCN, is dampened, further altering the organism's circadian behavior. The SCN's functional regression causes a reduction in rhythmic production.^18^ The SCN can send signals to peripheral organs through the nervous system's parasympathetic and sympathetic arms, which aids in their entrainment. A lack of circadian rhythmicity of neurotransmitters in the submandibular glands of aged mice has decreased the clock's ability to react to changes in peripheral tissue, which is consistent with this.^19^ It remains to be seen if this would result in greater phase dispersion of individual cells within individual cells within individuals, and how this will affect the aging process in particular tissues. The SCN establishes humoral patterns to many regions within the brain, which further synchronize peripheral tissue clocks. Via the pineal gland, the SCN regulates the normal rhythms of melatonin and the regular rhythms of glucocorticoids.^20^ The robustness of clock rhythmic production in aged peripheral tissues is likely to be affected by these aging‐related increases in melatonin expression. Both shifts may be due to a human's age‐related deterioration and a reduction in the human's circadian clock.^21^

The clock's work can be affected by the aging process. The amplitude of circadian clock oscillations has been shown to change in vitro when the extracellular matrix (ECM stiffness) is mechanically sensed.^22^ It needs to be seen if this finding converts into circadian clock activity in vitro.

## 5 THE CELLULAR CLOCK MACHINE AND AGING

**FIGURE 3:**
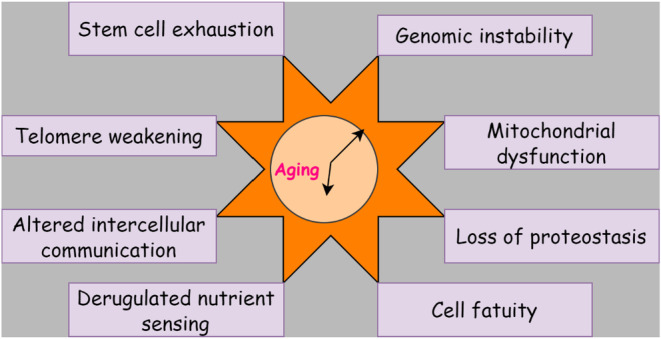
Impact of the circadian clock on aging‐loss of functional integrity.

Despite the above‐mentioned loss of functional integrity in the SCN and the possible decrease in synchronization signals with age, the molecular clockwork in most mammalian peripheral tissues maintain oscillation during aging, at least under the unaffected conditions. In most laboratory configurations, entrainment conditions are used (Figure 3).

To better understand the aging process, a more systematic analysis, with multiple core clock reporters at each core clock, or studies at the RNA and protein levels at different ages, may be needed.^23^ The central clockwork seems to be in good working order. With maturity, most peripheral tissues are found to be robustly oscillating under unaffected entrainment conditions. The ability of older tissues to react to changes in environmental entrainments is decreased. This means that if the robustness of signals downstream of the SCN is reduced, the peripheral clocks' ability to react to changes is in entrained cues.^24^

Aging is controlled by a number of mechanisms, and most of these systems are influenced by it. The influence of the circadian clock on the aging process is determined by the balance of circadian proteins. The most striking example of the impact of circadian clock proteins on aging is mice without BMAL1. Cry1, two double‐deficient mice have not shown signs of accelerated aging.^25^ After being exposed to non‐lethal levels of radiation, these mice may experience accelerated age‐related diseases. This points to a complicated relationship between the circadian clock and aging, which is influenced by various proteins and the circadian clock's activity. The idea that age can impair circadian clock activity has been recognized for decades.^26^ A major mechanism that prevents the accumulation of DNA damage is DNA repair. The precise mechanism by which SIRT1 and other Sirtuins family members function is still under investigation. SIRT1 is controlled by the circadian mechanism, which may play a role in genome integrity circadian regulation. Defects in genome integrity are often linked to a rise in the incidence of cancer.^27^

## 6 PERIPHERAL TISSUE AGING AND CIRCADIAN PHYSIOLOGY

**FIGURE 4:**
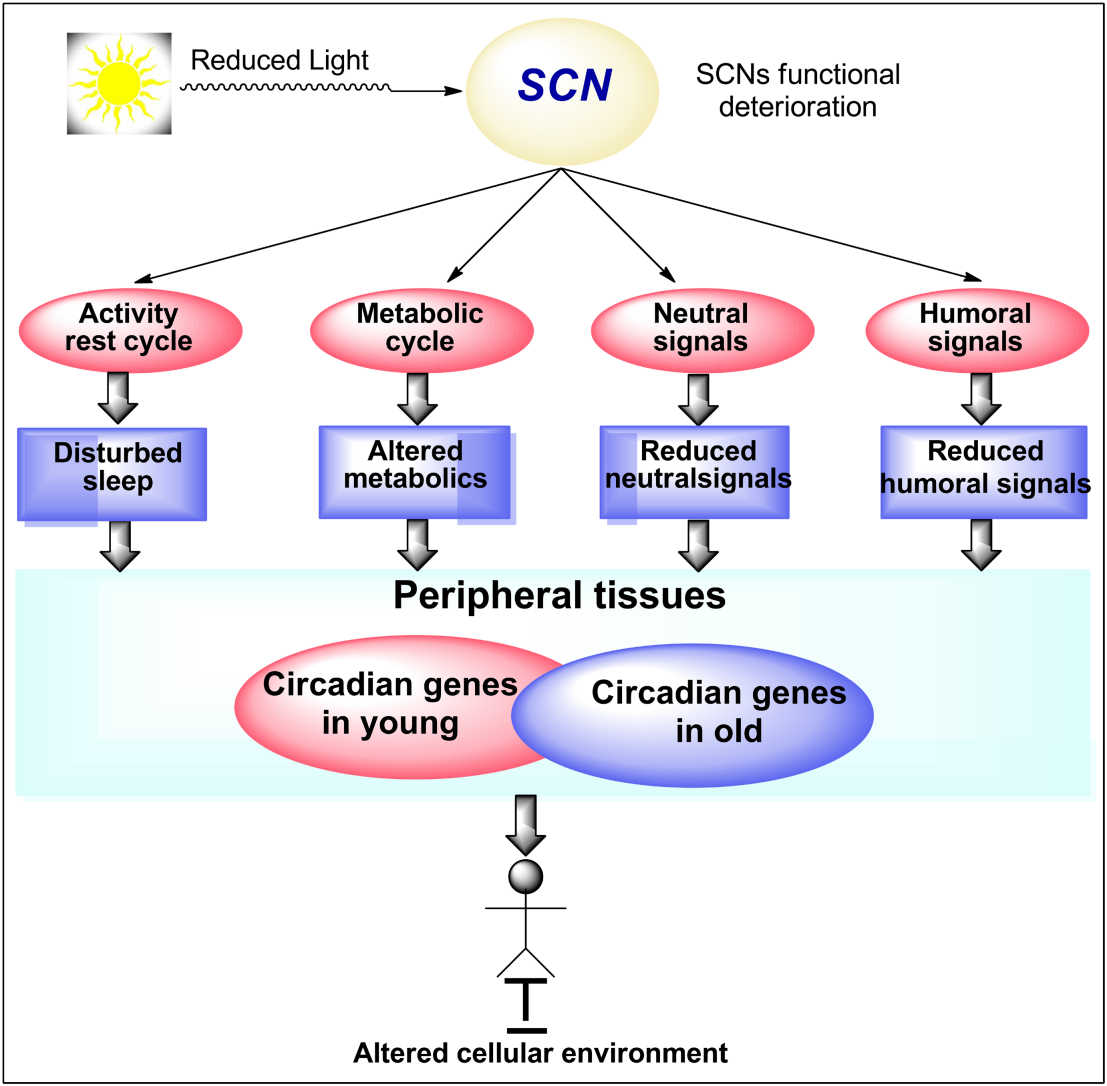
Effect of aging on peripheral tissues‐circadian physiology. SCN, supra chiasmatic nucleus.

The circadian clock controls both genes and pathways, as shown in Figure 4. Circadian production is tissue‐specific and includes a number of mechanisms and processes related to aging. Neurogenesis can be inhibited by disruptions in normal daily rhythms, such as those experienced during jetlag when the external world is out of sync with the circadian clock. The circadian clock in the cell is in control of the cell.^28^ Most immune cells have a strong circadian production and an oscillating clockwork. Many immune system roles, such as recruiting, diagnosis, and reaction to pathogens, are time‐dependent. The normal cycle of hematopoietic stem cells (HSCs) from bone marrow to tissues and back is controlled by the circadian clock.^29^ The peak of oxidation happens during the day (when mice are sleeping), followed by an increase in reactive oxygen species (ROS) levels. The night‐time is when DNA replication peaks (while mice are sleeping). This is to ensure that DNA replication takes place when ROS levels are high enough.^30^ Many genes involved in metabolic processes, for example, are expressed in a circadian manner in the liver. This daily variation most likely causes the liver metabolome to respond to daily feeding and nutrient supply cycles.^31^

All of these examples highlight the role of the circadian clock in controlling the many specific tasks carried out by various functions on a daily basis, all of which are essential for maintaining stable homeostasis. Among other factors, the circadian clock regulates intestinal permeability, body structure, and the immune response to gastrointestinal infections.^32^ The functional changes of the circadian network during the aging process can have an effect on the aging process.^33^ The question of whether the circadian clock influences the aging process by regulating its activity is crucial. A new model for aging's effect on aging's work has been proposed in some studies. The clock's normal rhythmic performance is reprogrammed in a variety of ways to control aging. Just a limited number of genes that are circadian in young tissue stay that way as they age.^34^ The stress‐related functions are invariably affected by the circadian production of newly developed age‐related circadian activity. When it comes to tissue‐specific pressures, such as the aging of young skeletal cells, the aging effect is more pronounced. Stress has an influence on the aging clock, as seen by the activity of the cells' circadian clock. Cell‐related stress‐response functions show the impact of the aging effect on stress‐response functions.^35^ It is possible that a reprogrammed circadian transcriptome in aged stem cells could hasten the aging process. In aged epidermal stem cells, DNA replication is delayed on a daily basis. Dietary restriction inhibits age‐related reprograming in the liver, epidermis, and muscle, and it also prevents both the delay in daily replicated DNA replication and the rise in oxidized DNA replication, at least in muscle.^36^

## 7 THE CIRCADIAN CLOCK AND AGING: MOLECULAR LINKS

The robustness of the structural pursuant circadian clockwork mechanism is reduced as the circadian input pathways, the SCN, and the output decidedly pathways deteriorate during the aging process. The circadian output of aged peripheral organs is reprogrammed, which most certainly contributes to the aging process. Sirtuins are a form of deacylase that needs NADNAD as a cofactor to work. Their operation is synchronized with the 24‐hour clock. The functions of three related sirtuins are primarily linked to the circadian clock: genetic and cytoplasmic SIRT1, mitochondrial SIRT3, and nuclear Sirt1. During aging, the actions of SIRT2 and Sirt3 have a mutual effect on each other. The work of the circadian clock and the actions of SRT1 are linked in a feedback loop that can be tracked back to the cell's energy state.^37^ In HSCs, SIRT3 activity is critical for preventing oxidative stress. In aged HSCs, its activity is suppressed, whereas upregulation enhances regenerative potential. However, it is unclear if the circadian clock is active in the SIRT3‐dependent modulation of HSC aging. The role of SIRT6 and the aging mechanism have yet to be identified by the circadian clockwork. The aging mechanism has been linked to its function in genome stability regulation. There is yet to be identified a clear relation between aging and the circadian clock. The clockwork was created to keep the human circadian clock from aging.^38^ Its purpose is to maintain telomeres by negatively regulating gene expression.

It is possible to have a longer lifespan in part due to male mice overexpressing a longer lifespan, but aging‐induced decline of the circadian clock is more likely. The aging process can be accelerated by clockwork.^39^ The mTOR is a serine/threonine kinase that is found in two functionally distinct signaling complexes in mammals. It functions as a vital core nutrient indicator. Via its function in behavioral rhythms, the mTOR clock influences the circadian clock in the SCN. In peripheral tissues, it may also lengthen the time and dampen the rate of oscillations.^40^ It is a direct target of S6K1, which causes BMAL1 to associate with the translational machinery, resulting in protein synthesis oscillations. It also has an effect on SCN's potential to be subject to a switched light/dark period. It acts as a suppressor of mTOR activity. It may also trigger an erratic circadian pattern in mice, which has an effect on their longevity and that of human's circadian rhythms.^41^ As AMP‐activated kinase (AMPK) is activated by low energy, it activates glycolysis and fatty acid oxidation to increase ATP levels. The interaction of the central clock machinery with AMPK is expected to play a role in aging. AMPK activation extends the lifetime of C. elegans and is thought to influence aging through the modulation of downstream pathways such as those with SIRT1 and CRYYY.^42^ It is worth noting that one popular anti‐aging strategy, dietary restriction (DR), affects all of the nutrient‐sensing signaling pathways listed above. The effects of DR and the circadian clockwork are molecularly related. DR's enhanced circadian entrainment mechanism could help to slow down the aging process. Improved dietary consumption during the aging period is a key factor in extending life expectancy. Because DR fails to extend the lifespan of Bmal2‐deficient mice, the lifespan of mice can be extended by modifying the response of the central clock gene Bmal1, which is necessary for the lifespan‐extending functions of DR.^43^

## 8 THE CIRCADIAN CLOCK IS A TIMEKEEPING DEVICE THAT KEEPS TRACK OF THE MOLECULAR LINKS TO AGING AND GENE DISRUPTION

In vivo aging has been related to depletions or mutations in core clock genes. Many of these mutations exhibit phenotypes that are closely linked to the aging mechanism. ClockD19 mice, for example, are vulnerable to being obese. This could help to understand why Bmal1/ and ClockE/e mutant mice have different lifespans. The increased arrhythmia in Clock D19 mice, on the other hand, raises the risk of cataracts and dermatitis in older mice.^44^ The disturbance of circadian rhythmicity caused by genetic deletion or mutation of core clock genes is related to the aging process. The physiological aging mechanism does not always fully conceal the aging process. However, it does play a part in the creation of some of the patterns linked to it.^45^

All of the three mouse models have lost their central clockwork rhythmicity in peripheral tissues. Gene‐specific roles, which may or may not include a role in the circadian clock, are likely to play a role in the creation of some of the pathologies seen in these mouse models. Rapatar, an mTOR inhibitor, could provide one potentially specific correlation between Bmal1 deficiency and treatment.^46^ In Bmal2/e mice, mTOR signaling is linked to a shorter lifespan. There are also no mechanistic developments into the ClockD19 and ClockE/e mouse models' shortened lifespan phenotypes. Mice with a faulty circadian clock show a faster loss in fertility and begin to lose soft tissues and experience kyphosis at the age of 12–14 months. Despite some phase dissonance between SCN and peripheral clocks, Cry1/ and Cry2/ mice live a normal lifespan. Although several mice have been confirmed to have a higher tumor frequency as they age, this is still inconclusive. In full darkness, the mice's behavior is arrhythmic, but they do not age prematurely (at least not within 18 months). The lifetime of mice with weak clockwork indicates some phase dissonance, indicating that the SCN and peripheral clocks are out of sync.^47^ Mice with mutations in various core clock genes show signs of premature aging and have shorter lifespans. In certain circumstances, these phenotypes do not seem to be related to improvements in circadian behavior or molecular circadian rhythmicity. This indicates that the individual clock genes' tissue‐specific roles are essential in the aging process.^48^ To better understand the tissue‐specific roles of the individual circadian clockwork components in the aging process, as well as their role in deciding longevity, further research is needed. There is no decrease in lifespan in mice as Bmal1 is depleted before regeneration rather than during development. A lack of core clock genes has been attributed to a number of aging symptoms. Early in postnatal development, BMAL1 appears to be necessary for certain processes. We really do not understand how loss of circadian clock genes causes premature aging.

The circadian clock is influenced by aging in part due to a reduction in light sensitivity and delivery to the SCN. The output of the circadian clock in peripheral tissues is reprogrammed as people age. At the molecular level, the circadian clock is linked to many aging‐related pathways. Understanding the molecular details of the aging‐related pathways that contribute to the functional impairment of SCN is important. The aging mechanism and the circadian clock's activity are inextricably linked.

Shifting work and modern lifestyles are linked to increased exposure to light at night, which raises the risk of contracting a variety of metabolic disorders, as well as cancer in humans and mice. The molecular information relating aging and the circadian clock work would need to be deciphered in future research. The objective is to find approaches that will allow for the maintenance of a well‐functioning circadian clock system as people age. It is just the beginning to figure out how circadian desynchronization could lead to these aging‐related molecular changes.

## 9 CONCLUSION

The circadian clock is an intrinsic, biological timekeeping system that regulates various physiological and behavioral processes in organisms, including the sleep–wake cycle, metabolism, and immune function. Over the years, research has revealed a close interconnection between the circadian clock and aging, as well as carcinogenesis – the process of cancer development. Understanding the molecular mechanisms that link circadian regulation, aging, and cancer has become a rapidly expanding field of study with profound implications for both basic science and potential therapeutic interventions. At the core of the circadian clock machinery are a set of genes that form transcription‐translation feedback loops. Two key transcription factors involved in these loops are BMAL1 (Brain and Muscle ARNT‐Like 1) and CLOCK (Circadian Locomotor Output Cycles Kaput). These proteins heterodimerize and bind to specific DNA sequences, leading to the activation of downstream clock‐controlled genes. The expression of these clock genes exhibits a 24‐hour rhythm, reflecting the cyclical nature of the circadian system.

The link between the circadian clock and cancer has been established through various studies. BMAL1 and CLOCK have been implicated not only in the regulation of cell cycle progression but also in the response to genotoxic stress, which can lead to DNA damage. The proper functioning of the circadian clock appears to act as a protective mechanism against the development of cancer by coordinating the repair of damaged DNA and controlling the cell cycle.

However, many questions remain unanswered in this complex interplay among the circadian clock, aging, and cancer. The precise functions of individual clock elements and their contributions to specific cellular processes are still areas of active investigation.

The potential therapeutic implications of targeting the circadian clock in the context of aging and cancer are also under exploration. If the molecular connections between the circadian clock and these processes can be fully unraveled, it may pave the way for the development of novel therapeutic strategies. For instance, modulating the circadian clock to enhance DNA repair or optimize the timing of cancer treatments could represent promising avenues for intervention.

In conclusion, the study of circadian control of aging and carcinogenesis represents a dynamic and rapidly evolving field with far‐reaching implications for human health. Continued research into the intricate molecular connections among the circadian clock, aging, and cancer holds the promise of not only expanding our fundamental understanding of these processes but also identifying new targets for therapeutic intervention in the treatment and prevention of age‐related diseases, including cancer.

## AUTHOR CONTRIBUTIONS

Conceptualization: Chandramouli and Ningaiah. Methodology: Chandramouli and Basavanna. Investigation: Basavanna and Chandramouli. Visualization: Chandramouli and Ningaiah. Supervision: Ningaiah. Writing of the original draft: Chandramouli. Writing of the review and editing: Chandramouli, Basavanna, and Ningaiah.

## FUNDING INFORMATION

There was no funding received for this work.

## CONFLICT OF INTEREST STATEMENT

The authors declare no conflict of interest.
